# Unilateral Endoscopic and Unilateral Biportal Endoscopic surgery for lumbar spinal stenosis: a systematic review and meta-analysis

**DOI:** 10.3389/fsurg.2025.1585783

**Published:** 2025-06-16

**Authors:** Kun Li, Zhibin Zhang, Jiangyu Ran, Liang Ma, Xiangyu Meng

**Affiliations:** ^1^ Graduate School of Xinjiang Medical University, Urumqi, China; ^2^ Minimally Invasive Spine Surgery, Sixth Affiliated Hospital of Xinjiang Medical University, Urumqi, China

**Keywords:** spinal stenosis, unilateral biportal endoscopic spine surgery, unilateral endoscopic spine surgery, laminectomy, unilateral laminotomy for bilateral decompression, endoscopy

## Abstract

**Objective:**

Lumbar spinal stenosis (LSS), the most frequently occurring degenerative spinal disease, significantly affects patient well-being. Preliminary clinical studies indicate favorable outcomes from unilateral biportal endoscopy (UBE) and unilateral endoscopy (UE) for managing LSS. This meta-analysis assessed the clinical effectiveness and safety profiles of unilateral laminotomy for bilateral decompression (ULBD) via these two minimally invasive endoscopic methods, aiming to establish evidence-based clinical recommendations.

**Materials and methods:**

A thorough examination of electronic databases was performed, encompassing PubMed, Cochrane Library, Web of Science, Embase, Medline, CNKI, WanFang, and VIP. Research assessing the clinical outcomes and complications of UBE-ULBD compared to UE-ULBD in the treatment of LSS was deemed suitable for inclusion. The outcome measures extracted comprised the Visual Analogue Scale (VAS), Oswestry Disability Index (ODI), duration of surgery, length of hospitalization, intraoperative blood loss, area of postoperative dural sac expansion, angle of ipsilateral facet joint resection, and occurrences of surgical complications.

**Results:**

Seventeen articles met inclusion criteria, encompassing one prospective cohort study, two case-control studies, and fourteen retrospective studies, involving 1, 457 total patients. The meta-analysis indicated that there were no statistically significant differences observed between the UBE and UE groups in terms of postoperative VAS scores for back and leg pain, as well as ODI scores at the intervals of 1 week, 3–6 months, and 6–12 months (*P* > 0.05). Nevertheless, the UBE methodology exhibited markedly reduced operative durations (*P* = 0.005) and enhanced postoperative expansion of the dural sac (*P* < 0.0001). Estimated intraoperative blood loss, hospitalization duration, complication rates, and ipsilateral facet joint resection angles were comparable between groups (*P* > 0.05).

**Conclusions:**

The meta-analysis indicates that the UBE technique exhibits similar long-term clinical efficacy, blood loss, duration of hospital stay, and rates of complications when compared to the UE technique. However, the UBE group exhibited shorter surgical duration and greater dural sac expansion area.

## 1 Introduction

LSS represents a common degenerative spinal condition, primarily attributed to progressive degenerative alterations, such as facet joint hypertrophy and ligamentum flavum thickening, which gradually narrow the spinal canal and subsequently compress the cauda equina and nerve roots (1, 2). Patients typically exhibit symptoms including pain in the lower back and legs, weakness, numbness, and classical intermittent claudication. Given the aging global population, the incidence of LSS is rising, considerably affecting the physical health, mental well-being, and economic status of middle-aged and elderly individuals (3). Conservative treatment can alleviate symptoms in mild-to-moderate stenosis, whereas early surgical intervention is recommended for severe cases to improve clinical outcomes. With advanced understanding of LSS, unilateral decompression alone is often considered insufficient for patients with bilateral symptoms. To address this limitation, Spetzger (4) first introduced the ULBD technique in 1997, which has since demonstrated favorable clinical outcomes. However, both unilateral and bilateral decompression techniques inherently carry risks of compromising spinal stability (5), potentially leading to postoperative complications such as chronic low back pain and paraspinal muscle atrophy in severe cases (3). With increasing adoption of minimally invasive concepts, UE and UBE spine surgery techniques have progressively evolved from the ULBD approach. Compared to conventional open surgery, both minimally invasive techniques offer distinct advantages, including smaller incisions, reduced intraoperative blood loss, and faster postoperative recovery. Gao et al. (6) retrospectively analyzed PE-TLIF and MIS-TLIF procedures, demonstrating significantly lower postoperative inflammatory marker levels (CK and CRP) in the PE-TLIF group on postoperative day 3 (*P* < 0.05). These findings confirmed enhanced musculoskeletal preservation with endoscopic techniques. Orthopedic trauma research further supports these findings. A study on humeral shaft fracture fixation objectively quantified surgical invasiveness through peripheral inflammatory markers (CRP, IL-6), showing significantly higher inflammatory indices with open reduction internal fixation (ORIF) compared to closed reduction (7). These consistent findings across surgical specialties robustly validate the tissue-sparing advantages of minimally invasive approaches, encouraging their adoption by orthopedic surgeons. Despite numerous clinical trials reporting outcomes of UE-ULBD and UBE-ULBD (8, 9), a systematic comparison of their efficacy and safety profiles remains lacking. Current controversies primarily involve three key aspects:(1) Whether neurological improvement differs significantly between the two minimally invasive techniques; (2) Whether the biportal UBE approach offers superior advantages over the UE technique in reducing intraoperative blood loss or complication risk; (3) Debates regarding perioperative outcomes, particularly hospitalization duration and operative time. This meta-analysis aims to synthesize existing clinical evidence to address these critical questions: (1) Comparing postoperative clinical outcomes between UBE and UE at standardized follow-up intervals (1 week, 3–6 months, and 6–12 months), focusing primarily on functional improvement (ODI) and pain relief (VAS scores); (2) Evaluating safety profiles by analyzing intraoperative blood loss, complication rates, and other adverse events; (3) Quantitatively assessing perioperative parameters. The findings will provide evidence-based recommendations to guide spine surgeons in selecting optimal minimally invasive approaches and establish standardized evaluation metrics for future research.

## 2 Data and methods

### 2.1 Literature retrieval strategy

#### 2.1.1 Retrievers

Kun Li, Zhibin Zhang.

#### 2.1.2 Databases

The following databases were searched electronically: VIP, WanFang, PubMed, Cochrane Library, EMBASE, and China National Knowledge Infrastructure (CNKI).

#### 2.1.3 Search terms

Keywords: “lumbar spinal stenosis”, “Unilateral Biportal Endoscopic Spine Surgery”, “Unilateral Endoscopic Spine Surgery”, “Unilateral Laminotomy for Bilateral Decompression”.

#### 2.1.4 Timeframe

Studies comparing UBE and UE treatments for LSS were retrieved from the inception of each database up to November 2024.

#### 2.1.5 Search strategy

An exhaustive electronic literature review was performed using PubMed, Cochrane Library, EMBASE, CNKI, WanFang, and VIP databases, encompassing publications up to November 2024. Search strategies combined subject headings and free-text terms using Boolean logic operators (“AND”, “OR”, “NOT”). Inclusion criteria restricted studies to English and Chinese human clinical trials.

### 2.2 Inclusion and exclusion criteria

#### 2.2.1 Inclusion criteria

(1) Study inclusion criteria comprised: Participants diagnosed with LSS; (2) Studies designed as randomized controlled trials (RCTs) or retrospective studies comparing UBE and UE; (3) Intervention groups underwent UBE, whereas controls received UE; (4) Outcomes included: ① operative duration; ② estimated blood loss; ③ length of hospitalization; ④ complications; ⑤ intraoperative fluoroscopy usage; ⑥ modified Macnab criteria; ⑦ back pain VAS; ⑧ leg pain VAS; ⑨ ODI; ⑩ postoperative dural sac expansion; ⑪ ipsilateral facet joint resection angle; (5) Minimum follow-up duration between 3 and 6 months.

#### 2.2.2 Exclusion criteria

(1) Studies with fewer than 20 cases; (2) Literature reviews, case reports, or conference abstracts; (3) Incomplete original data; (4) Duplicate publications.

### 2.3 Data extraction

Reference management software (EndNote X9, Thomson Scientific, USA) was used to manage retrieved articles. Two researchers independently screened titles and abstracts to exclude clearly irrelevant articles. Subsequently, researchers independently applied inclusion and exclusion criteria, screened remaining studies, and reviewed selected full-text articles. Two investigators independently evaluated study quality and extracted relevant data, resolving discrepancies via consultation with a third investigator. Corresponding authors were contacted when necessary to obtain supplementary data.

### 2.4 Literature quality assessment

The included studies were evaluated for quality by two separate reviewers. The Newcastle-Ottawa Scale (NOS) was used to assess the quality of cohort and case-control studies. Studies were considered high-quality if their scores were 7 or higher, moderate-quality if their scores were 5–6, and low-quality if their scores were less than 5. We used Review Manager 5.3 to evaluate the methodological quality of RCTs according to a set of criteria that included things like blinding of participants and researchers, blinding of outcome assessment, completeness of outcome data, selective reporting, and random sequence creation, among other things. There were visual representations of the evaluations of the study's quality. A third reviewer was brought in to settle disagreements over the results of the evaluation.

### 2.5 Outcome indicators

(1) Baseline characteristics: author, publication year, geographic region, study design, sample size, patient age, clinical presentation, surgical interventions, and follow-up durations. (2) Primary outcomes: ① operative time; ② estimated intraoperative blood loss; ③ hospitalization duration; ④ complication rates; ⑤ intraoperative fluoroscopy frequency; ⑥ modified Macnab criteria; ⑦ VAS back pain scores; ⑧ VAS leg pain scores; ⑨ ODI scores; ⑩ dural sac expansion area; ⑪ ipsilateral facet joint resection angle.

### 2.6 Statistical analysis

Statistical analysis was performed using Review Manager 5.3 software (Nordic Cochrane Centre). Dichotomous variables were reported as risk ratios (RR), while continuous data measured uniformly were expressed as mean differences (MD). Results included 95% confidence intervals (CI). Heterogeneity was evaluated using I^2^ statistics and associated *p*-values; a fixed-effects model was utilized for analyses with low heterogeneity (I^2^ < 50%, *p* > 0.1). Sensitivity analyses assessed the robustness of results by sequentially omitting individual studies. Funnel plots examined potential publication bias. Statistical significance was set at *p* ≤ 0.05.

## 3 Results

### 3.1 Literature search and screening process

**Figure 1:**
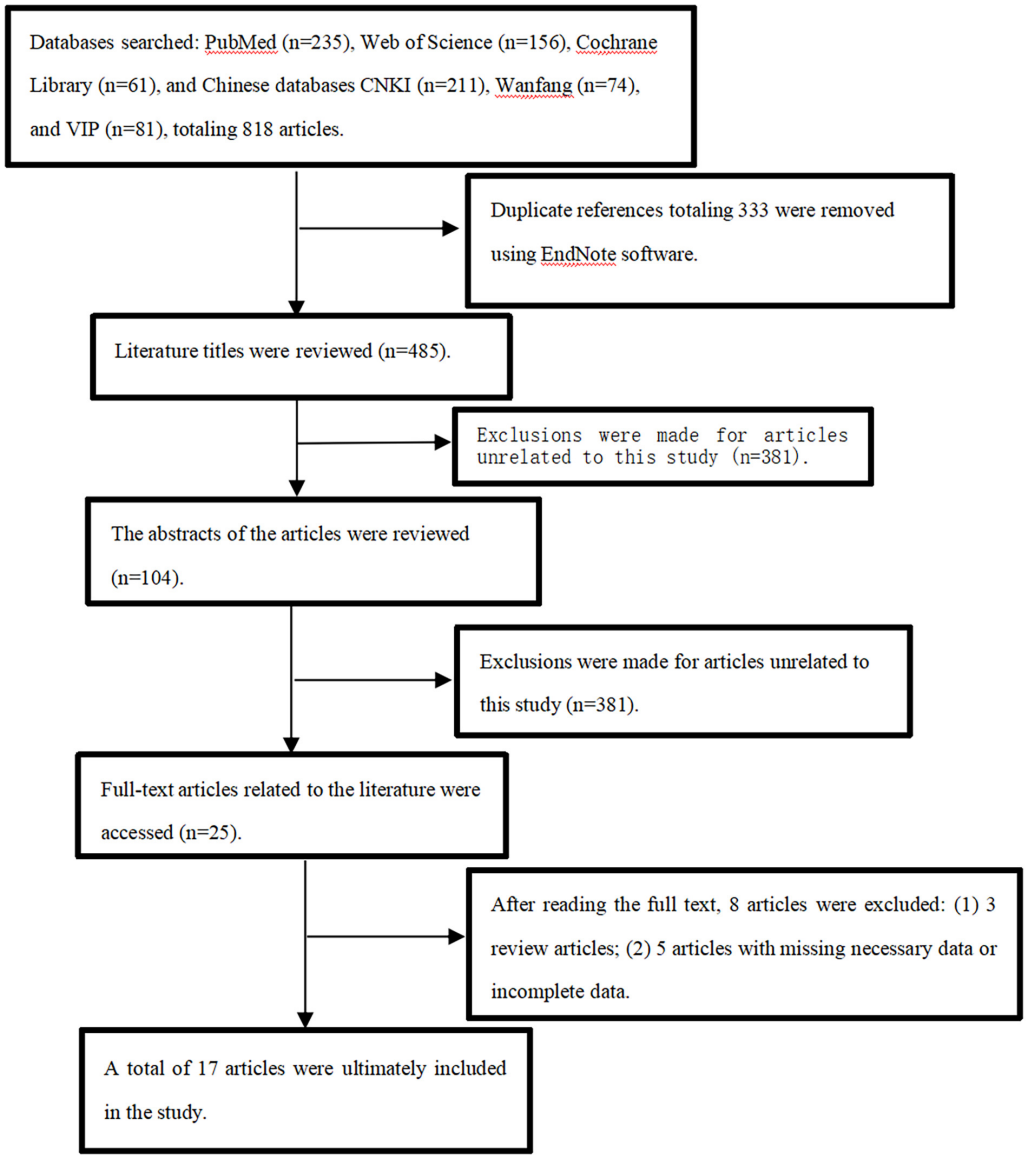
Literature screening process and results.

Beginning with PubMed (235 articles), Web of Science (156 articles), the Cochrane Library (61 articles), CNKI (211 articles), Wanfang (74 articles), and VIP (81 articles) were the initial databases searched. Two prospective cohorts and fifteen retrospective case-controls met the inclusion criteria after extensive screening; together, they included 1, 457 patients with LSS. Figure 1 shows the screening approach and outcomes in detail. Table 1 summarizes the key features of the studies that were considered. Table 2 presents the quality assessment of the included literature.

**Table 1:** Basic characteristics of the 17 included studies.

Author	Country	Design	Sample (M/F)		Age (year)		Disease	Segment		Follow/M	Outcome
			UBE	UE	UBE	UE		UBE	UE		
He et al. (13)	China	Retrospective study (RS)	20/13	15/17	67.72 ± 8.99	62.50 ± 8.37	LSS	L3-4: 6 L4-5: 18 L5-S1: 9	L3-4: 5 L4-5: 17 L5-S1: 10	12	①②③④⑥⑦⑧⑨⑩
Cheng et al. (10)	China	RS	107/25	62/19	61.3 ± 6.9	62.3 ± 6.4	LSS	L2-L3: 2 L3-4: 14 L4-5: 106 L5-S1: 10	L2-L3: 4 L3-4: 11 L4-5: 58 L5-S1: 8	12	①③④⑦⑨
Bi et al. (18)	China	RS	40/36	34/37	59.8 ± 6.7	59.3 ± 5.6	LSS	L3-4: 11 L4-5: 36 L5-S1: 29	L3-4: 10 L4-5: 34 L5-S1: 27	18	①④⑥⑦⑧⑨⑩
Xu et al. (26)	China	RS	19/22	26/31	64.9 ± 7.7	65.5 ± 11.8	LSS	L3-4: 35 L4-5: 5 L5-S1: 1	L3-4: 51 L4-5: 5 L5-S1: 1	12	①④⑥⑦⑨
Xu and Xu (25)	China	RS	18/14	10/12	65.81 ± 10.63	65.14 ± 10.53	LSS	L3-4: 4 L4-5: 21 L5-S1: 7	L3-4: 3 L4-5: 13 L5-S1: 6	6	①③④⑦⑧⑨⑩⑪
Wang and Xu (22)	China	RS	6/4	5/5	64.10 ± 8.08	64.9 ± 8.23	LSS	–	–	6	①③④⑥⑦⑧⑨⑩
Heo et al. (14)	Korea	RS	15/22	11/16	66.7 ± 9.4	67.3 ± 9.9	LSS	–	–	12	①②③④⑦⑧⑨⑩⑪
Wang et al. (13)	China	RS	13/10	13/12	61.52 ± 8.09	59.24 ± 4.11	LSS	L4-5: 12 L5-S1: 11	L4-5: 15 L5-S1: 10	6–12	①②③④⑦⑨
Hwang et al. (16)	Korea	Prospective study	24/21	11/21	65.3 ± 6.8	66.8 ± 9.1	LSS	–	–	12	①③④⑥⑦⑧⑨⑩
Hu et al. (20)	China	RS	20/22	16/24	63.2 ± 7.6	63.5 ± 7.5	LSS	L3-4: 9 L4-5: 28 L5-S1: 5	L3-4: 9 L4-5: 29 L5-S1: 2	6	①③④⑦⑧⑨⑩
Xu et al. (24)	China	RS	52/58	31/35	65.81 ± 10.63	65.14 ± 10.53	LSS	L3-4: 8 L4-5: 82 L5-S1: 32	L3-4: 4 L4-5: 49 L5-S1: 17	18	①⑥⑦⑧⑨
Hua et al. (15)	China	Case control study	15/21	14/22	64.1 ± 11.3	63.9 ± 12	LSS	L3-4: 9 L4-5: 24 L5-S1: 4	L3-4: 7 L4-5: 29 L5-S1: 2	12	①②③④⑥⑦⑧⑨
Wu et al. (17)	China	Prospective study	16/16	13/16	56.7 ± 8.9	57.3 ± 10.9	LSS	L3-4: 1 L4-5: 27 L5-S1: 7	L2-L3: 1 L3-4: 7 L4-5: 29 L5-S1: 2	12	①②③④⑥⑦⑧⑨
Cheng et al. (12)	China	RS	12/27	14/24	69.08 ± 7.23	69.45 ± 7.28	LSS	L3-4: 6 L4-5: 18 L5-S1: 15	L3-4: 5 L4-5: 19 L5-S1: 14	12–36	①②③④⑤⑥⑦⑧⑨
Han et al. (19)	China	RS	9/14	12/14	58.18 ± 21.03	52.5 ± 19.16	LSS	L3-4: 3 L4-5: 10 L5-S1: 10	L3-4: 2 L4-5: 9 L5-S1: 18	12–20	①③④⑦⑧⑨⑩
Tan et al. (21)	China	RS	11/11	17/12	62.45 ± 7.44	61.59 ± 8.79	LSS	L3-4: 5 L4-5: 17 L5-S1: 7	L3-4: 5 L4-5: 11 L5-S1: 6	12	①④⑤⑥⑦⑧⑨
Wang et al. (23)	China	RS	16/14	15/15	60.33 ± 2.38	60.37 ± 2.39	LSS	–	–	6	①③④⑦⑧⑨⑩

① Operation time; ② Estimated blood loss; ③ Hospitalization time; ④ Complications; ⑤ Intraoperative fluoroscopy; ⑥ Modified Macnab Criteria; ⑦ Back-VAS score; ⑧ Leg-VAS score; ⑨ ODI score; ⑩ Dural expansion; ⑪ Ipsilateral facet joint resection angle.

**Table 2:** Literature quality assessment.

Author	Selection of the study population	Establishing comparability	Outcome measurement	Score	Quality rating
He et al. (13)	★★	★★	★★	6	Medium quality
Cheng et al. (10)	★	★★★	★★	6	Medium quality
Bi et al. (18)	★★★	★	★★★	7	High quality
Xu et al. (26)	★★	★	★★	5	Medium quality
Xu and Xu (25)	★★	★★	★★	6	Medium quality
Wang and Xu (22)	★★	★★	★	5	Medium quality
Heo et al. (14)	★★★	★★	★★	7	High quality
Wang et al. (13)	★★	★★★	★★	7	High quality
Hwang et al. (16)	★★	★★★	★★★	8	High quality
Hu et al. (20)	★★★	★★	★	6	Medium quality
Xu et al. (24)	★★	★★★	★	6	Medium quality
Hua et al. (15)	★★	★	★★★	6	Medium quality
Wu et al. (17)	★★★	★★	★★★	8	High quality
Cheng et al. (12)	★★	★	★★	5	Medium quality
Han et al. (19)	★★	★★	★★	6	Medium quality
Tan et al. (21)	★★	★★★	★	6	Medium quality
Wang et al. (23)	★★★	★★	★★	7	High quality

### 3.3 Meta-analysis results

#### 3.3.1 Postoperative scoring criteria

To evaluate differences in VAS and ODI scores after surgery, groups were classified based on the timing of postoperative assessments.

##### 3.3.1.1 Back pain VAS scores

###### 3.3.1.1.1 Scores

**Figure 2:**
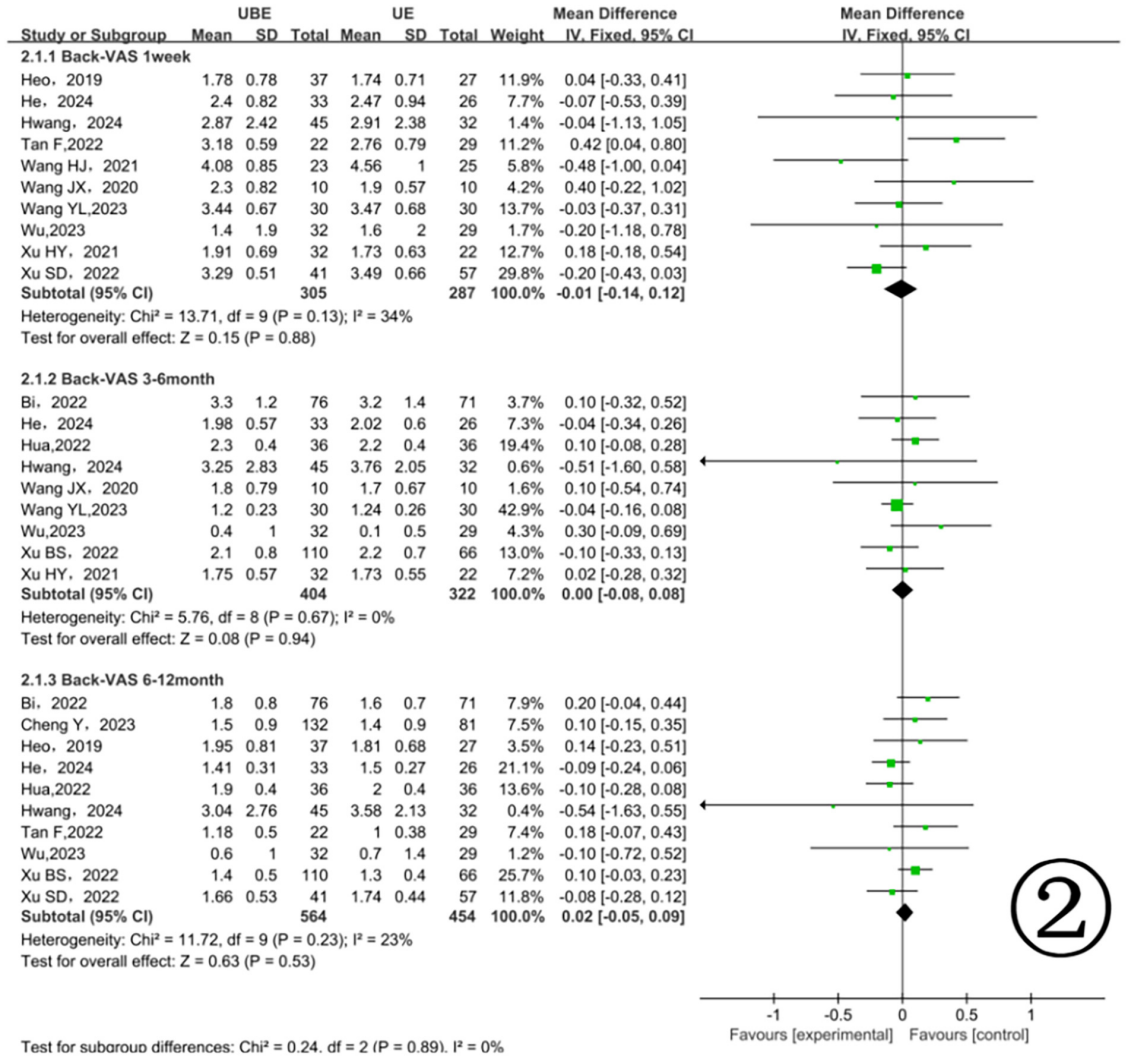
Comparison of back pain VAS scores between the two groups.

During the first postoperative week, 10 studies involving 592 patients reported back pain VAS scores (11, 13, 14, 16, 17, 21–26) (Figure 2). There was no significant difference between the two groups (MD = −0.01, 95% CI: −0.14, 0.12, *p* = 0.88). At 3–6 months postoperatively, 9 studies involving 726 patients reported back pain VAS scores (13, 14–18, 22–24), again showing no significant difference (MD = 0.00, 95% CI: −0.08, 0.08, *p* = 0.94). At 6–12 months postoperatively, 10 studies involving 1, 018 patients (10, 13–18, 21, 24, 26) similarly showed no significant difference (MD = 0.02, 95% CI: −0.05, 0.09, *p* = 0.53) (Figure 2).

##### 3.3.1.2 Leg pain VAS scores

**Figure 3:**
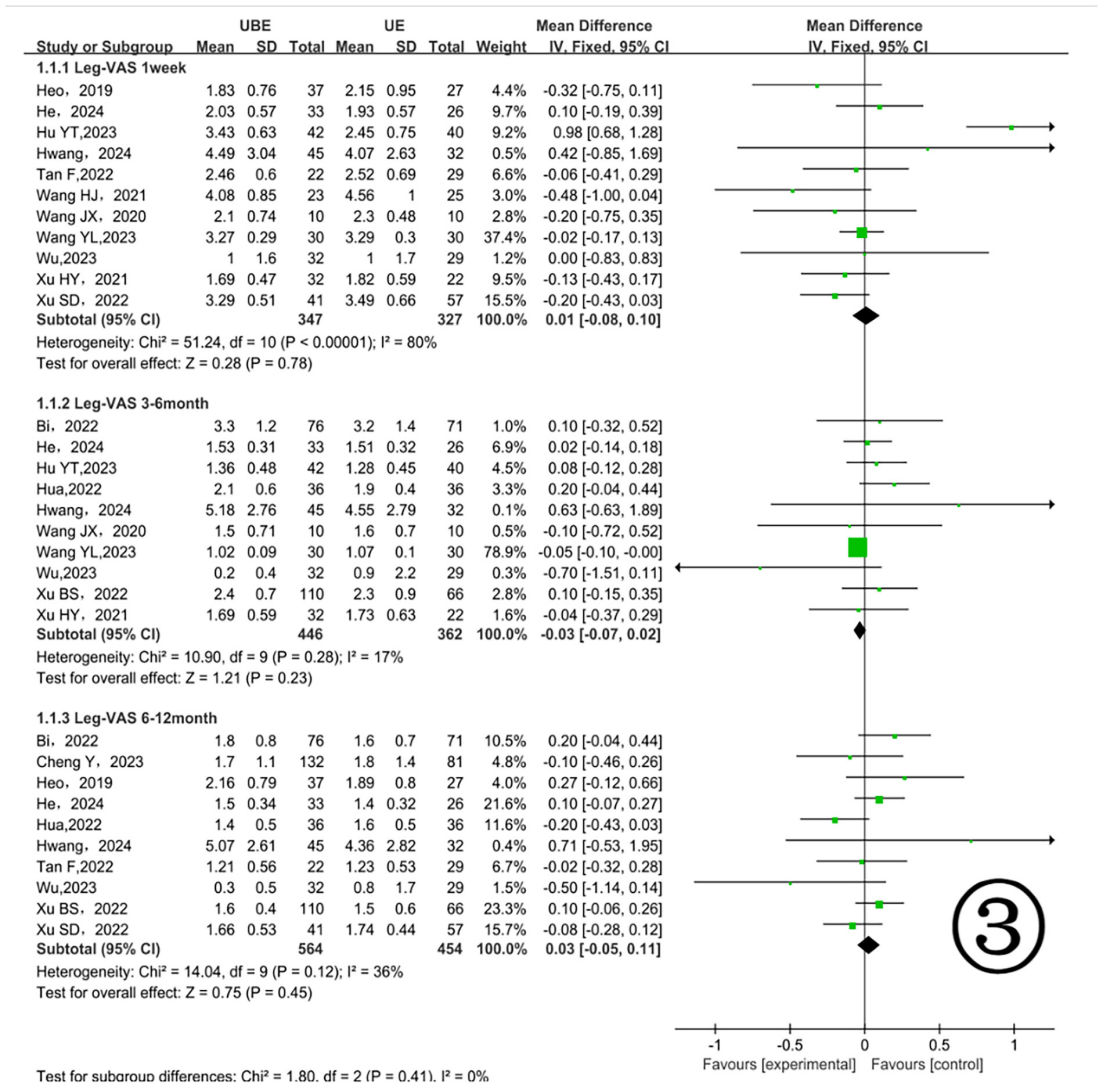
Comparison of leg pain VAS scores between the two groups.

At one week postoperatively, 11 studies involving 674 patients (11, 13, 14, 16, 17, 20–23, 25, 26) reported leg pain VAS scores (Figure 3), with no significant differences [MD = 0.01, 95% CI (−0.08, 0.10) *P* = 0.78]. From 3 to 6 months postoperatively, 10 studies with 808 patients (13, 15–18, 20, 22–25) were analyzed, showing no significant differences [MD = 0.03, 95% CI (−0.07, 0.02) *P* = 0.23]. Between 6 and 12 months postoperatively, 10 studies involving 1, 018 patients (10, 13–18, 21, 24, 26) again demonstrated no significant differences [MD = 0.03, 95% CI (−0.05, 0.11) *P* = 0.45] (Figure 3).

##### 3.3.1.3 ODI scores

**Figure 4:**
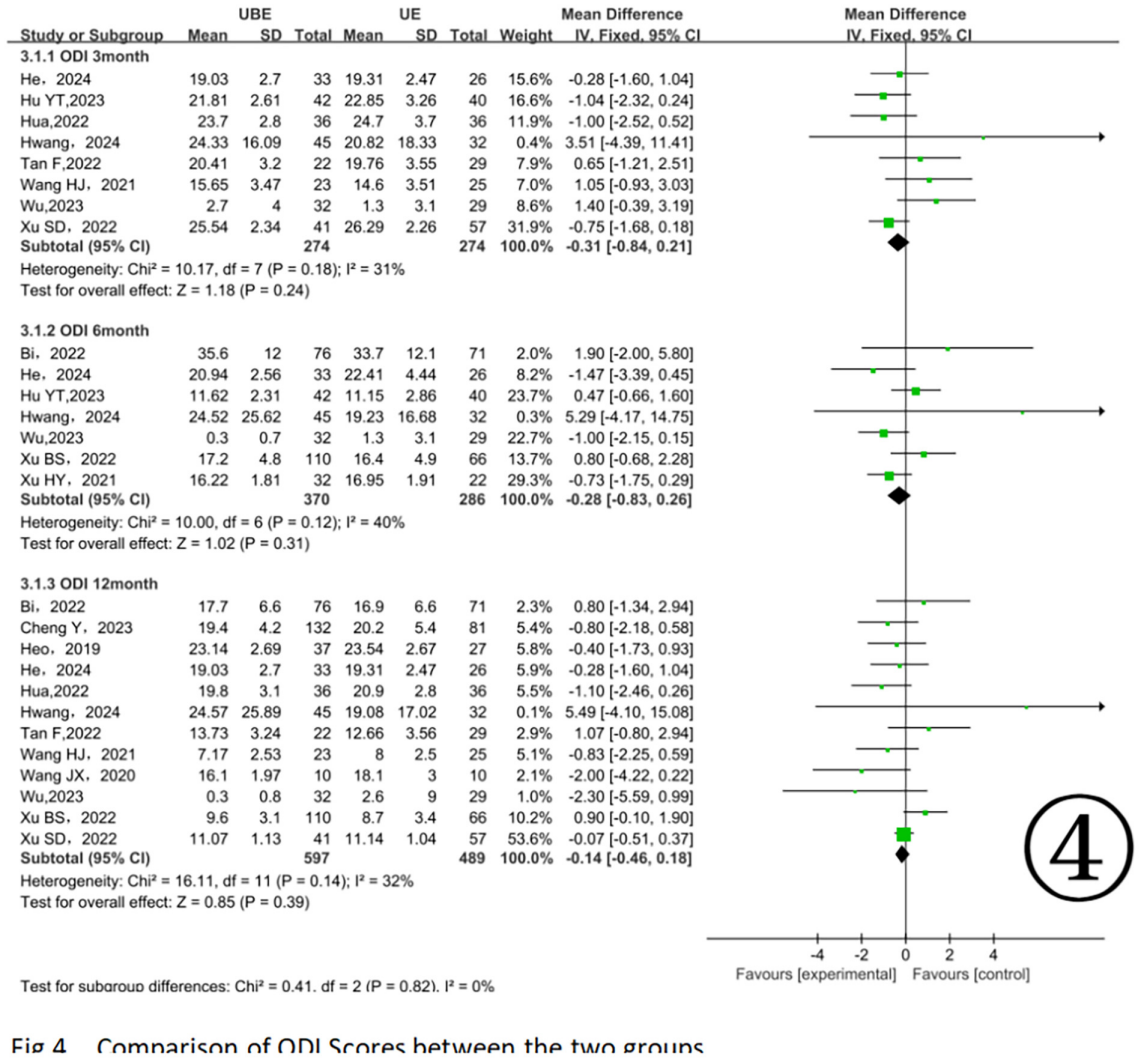
Comparison of ODI scores between the two groups.

At 3 months postoperatively, 8 studies involving 548 patients (11, 13, 15–17, 20, 21, 26) showed no significant differences between groups [MD = −0.31, 95% CI (−0.84, 0.21) *P* = 0.24]. Between 3 and 6 months postoperatively, 7 studies involving 656 patients (13, 16, 17, 20, 24, 25) indicated no significant differences [MD = −0.28, 95% CI (−0.83, 0.26) *P* = 0.31]. At 12 months postoperatively, 12 studies with 1, 086 patients (10, 11, 13–18, 21, 22, 24, 26) similarly revealed no significant differences [MD = −0.14, 95% CI (−0.46, 0.18) *P* = 0.39] (Figure 4).

#### 3.3.2 Parameter indicators

##### 3.3.2.1 Operation time

**Figure 5:**
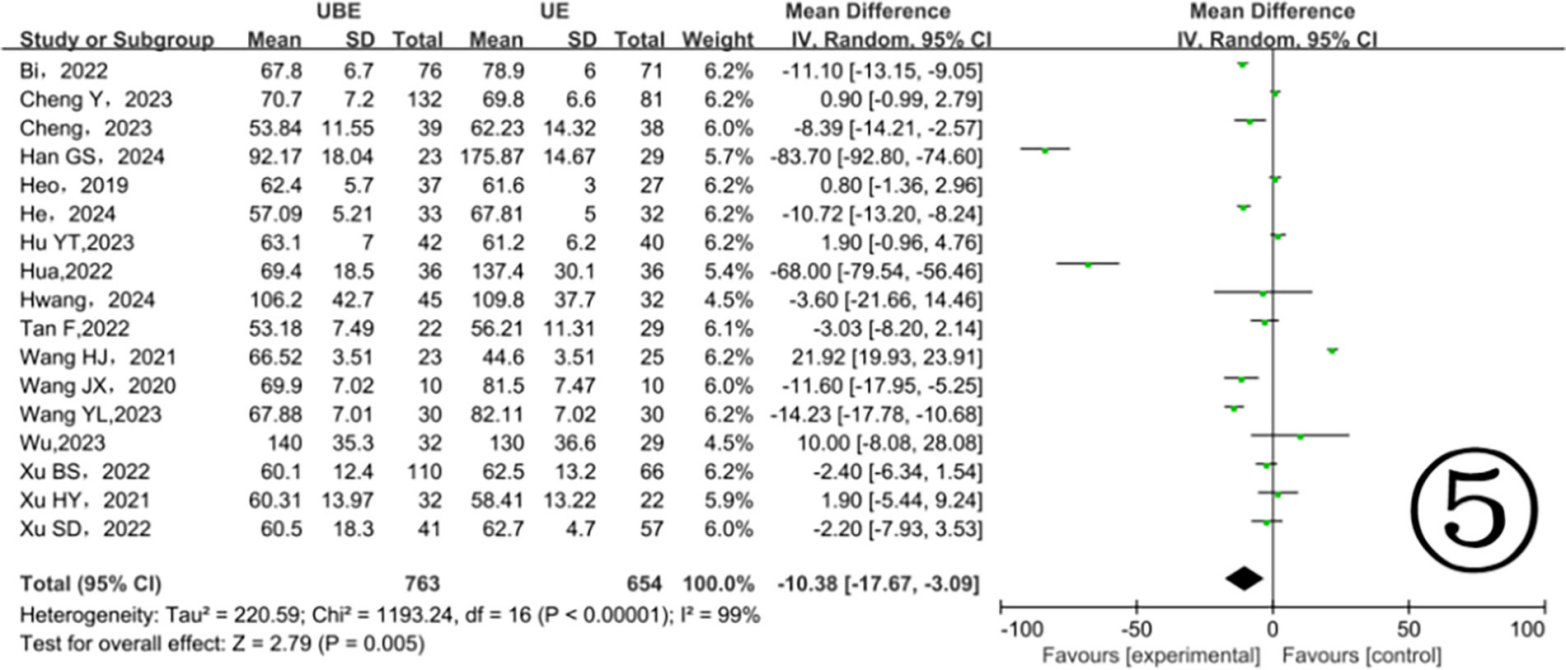
Comparison of operation time between the two groups.

**Figure 6:**
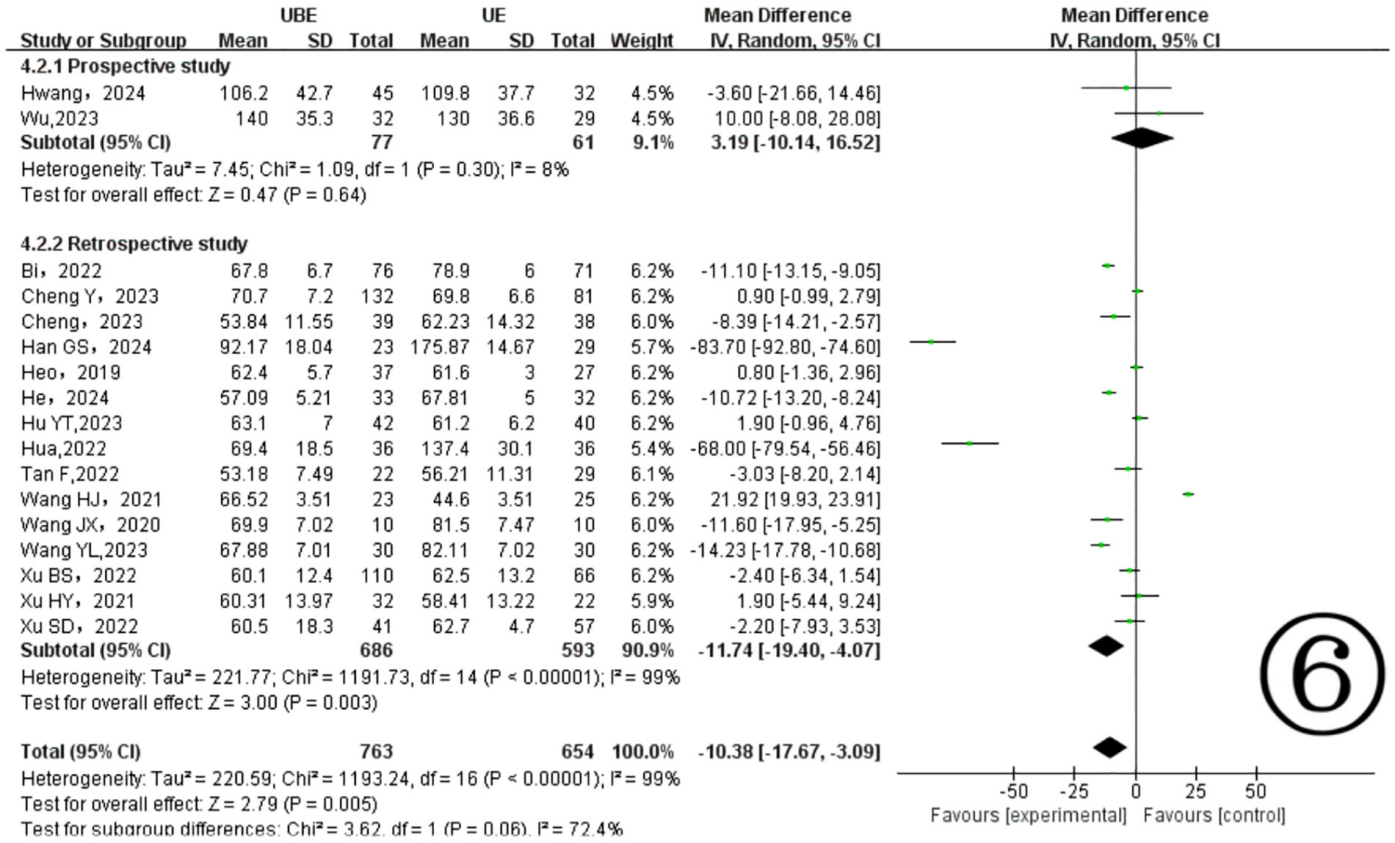
Subgroup analysis of the operation time.

Seventeen included studies (10–26) provided data on surgical durations, demonstrating notable heterogeneity (*P* < 0.00001, I^2^ = 99%). The results showed the UBE technique significantly reduced surgical duration compared to UE (MD = −10.38, 95% CI: −17.67 to −3.09, *P* = 0.005) (Figure 5). Subgroup analysis identified different heterogeneity levels: prospective studies indicated low heterogeneity (*P* = 0.30, I^2^ = 8%), whereas retrospective studies showed considerable heterogeneity (*P* < 0.00001, I^2^ = 99%) (Figure 6).

##### 3.3.2.2 Estimated blood loss

**Figure 7:**
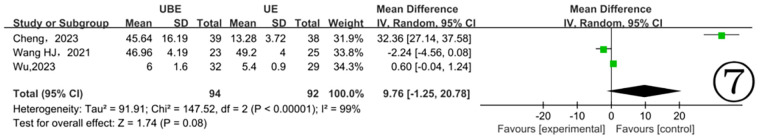
Comparison of estimated blood loss between the two groups.

Three studies (11, 12, 17) reported estimated intraoperative blood loss, with significant heterogeneity detected (*P* < 0.00001, I^2^ = 99%). The meta-analysis, using a random-effects model, found no statistically meaningful difference between UBE and UE groups (MD = 9.76, 95% CI: −1.25–20.78, *P* = 0.08) (Figure 7).

##### 3.3.2.3 Hospitalization time

**Figure 8:**
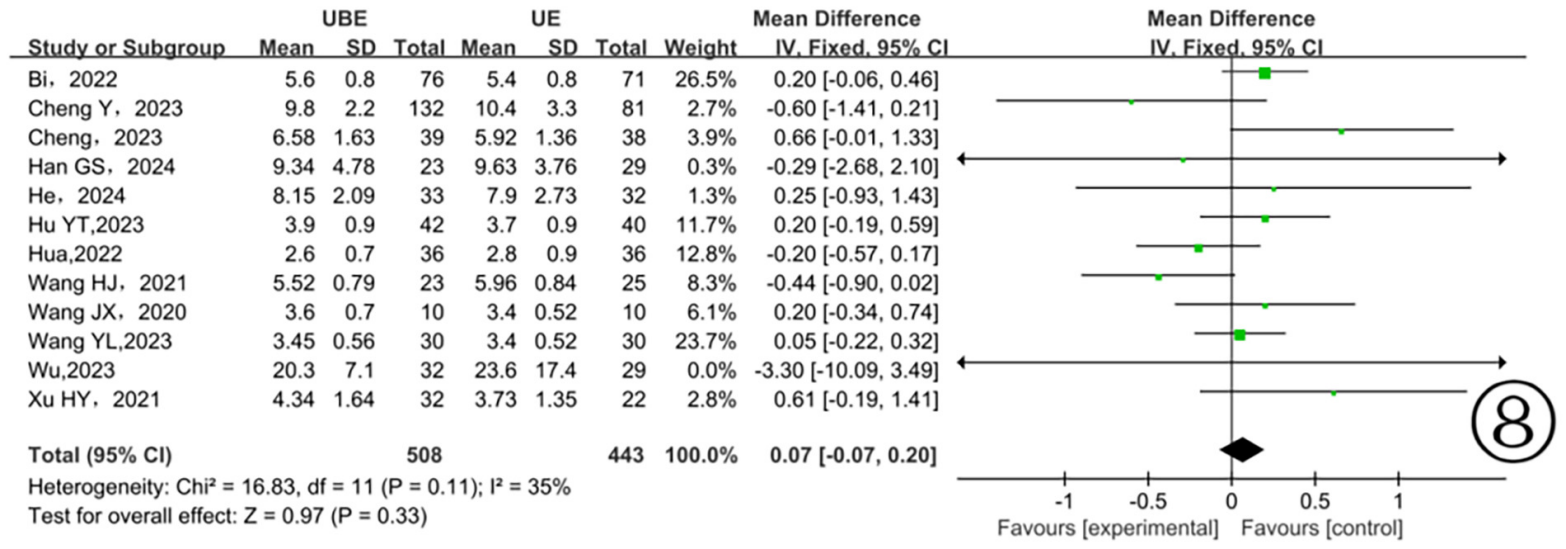
Comparison of hospitalization time between the two groups.

Data from eleven studies (10–13, 15, 17–20, 22, 23, 25) concerning hospital stay showed moderate and clinically acceptable heterogeneity (*P* = 0.11, I^2^ = 35%). Application of a fixed-effects model indicated no significant differences between the two surgical approaches (*P* = 0.33) (Figure 8).

##### 3.3.2.4 Complications

**Figure 9:**
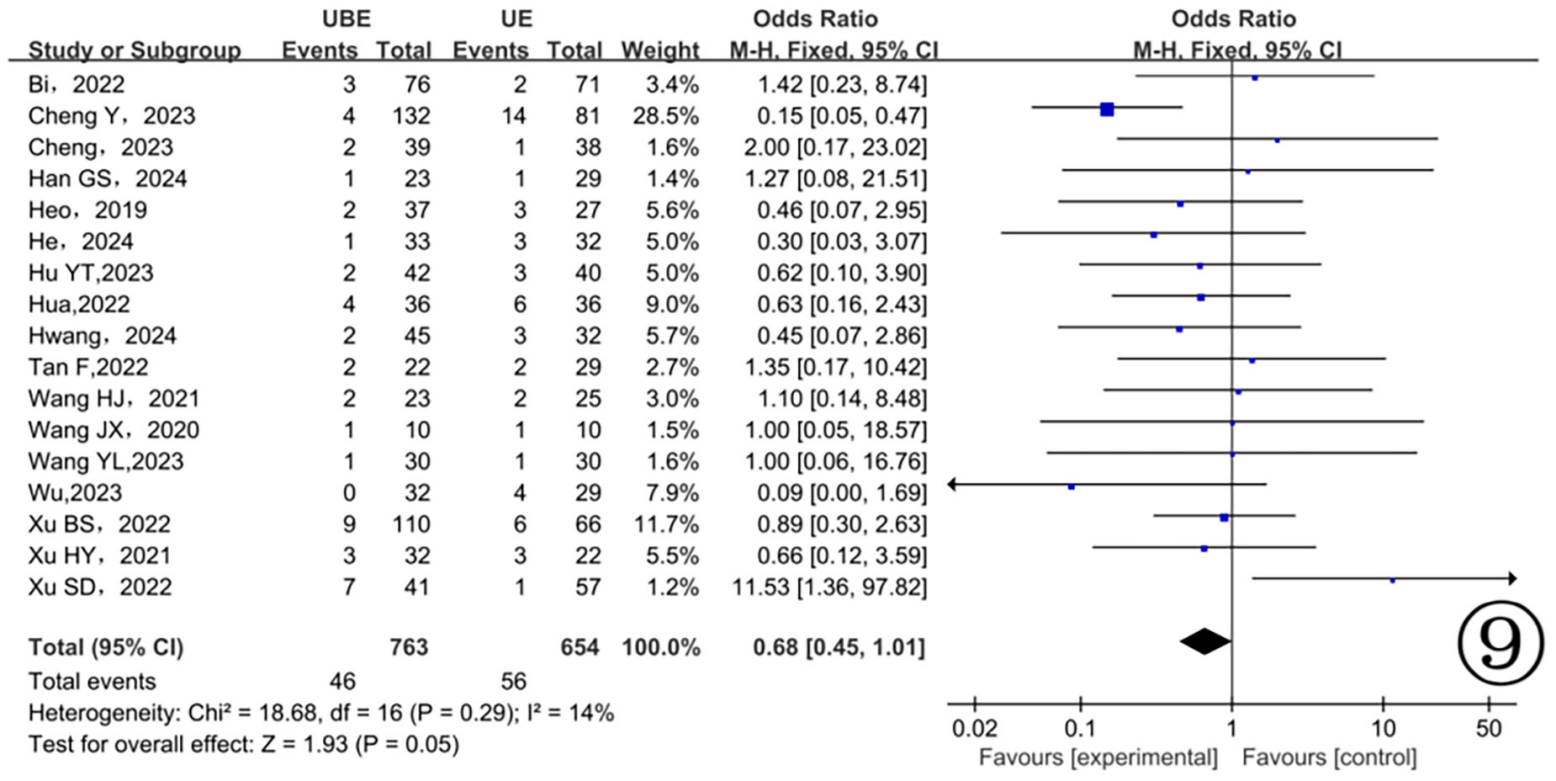
Comparison of complications between the two groups.

Postoperative complications were recorded in seventeen studies (10–26), demonstrating minimal heterogeneity (*P* = 0.27, I^2^ = 15%). Analysis using a fixed-effects model revealed no statistically significant differences between the two groups regarding postoperative complications (*P* = 0.08) (Figure 9).

##### 3.3.2.5 Dural expansion

**Figure 10:**
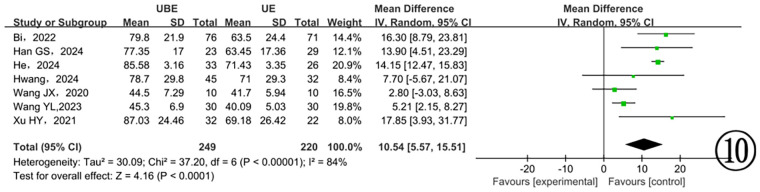
Comparison of dural expansion between the two groups.

Seven studies (13, 16, 18, 19, 22, 23, 25) provided data on dural sac expansion, revealing substantial heterogeneity (*P* < 0.00001, I^2^ = 84%). Employing a random-effects model, the analysis identified a significant difference favoring the UBE approach with enhanced postoperative dural expansion (*P* < 0.0001) (Figure 10).

##### 3.3.2.6 Ipsilateral facet joint resection angle

**Figure 11:**
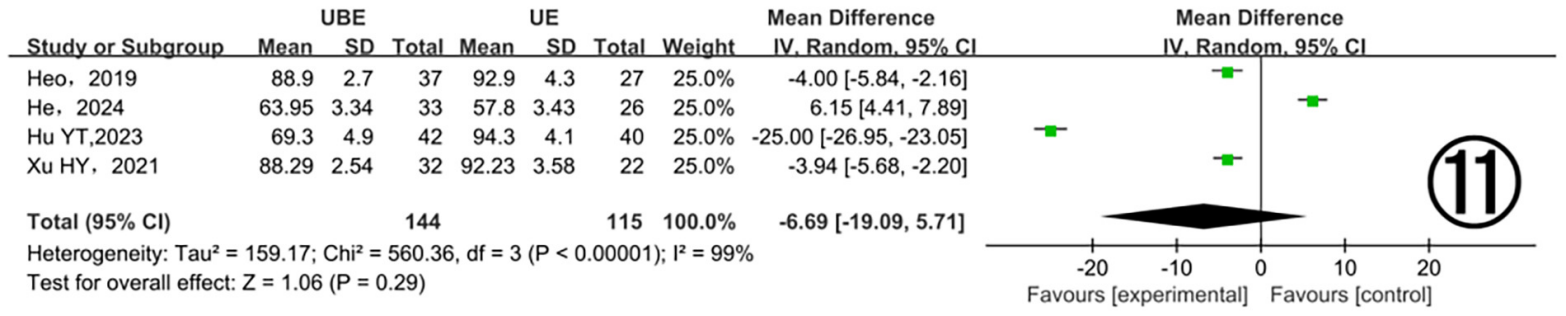
Comparison of ipsilateral facet joint resection angle between the two groups.

Four studies (13, 16, 20, 25) examined ipsilateral facet joint resection angles, demonstrating significant heterogeneity (*P* < 0.00001, I^2^ = 99%). Analysis using a random-effects model did not detect a significant difference between the two groups (MD = −6.69, 95% CI: −19.09–5.71, *P* = 0.29). The considerable heterogeneity observed warrants additional exploration of underlying causes (Figure 11).

### 3.4 Publication bias analysis

**Figure 12:**
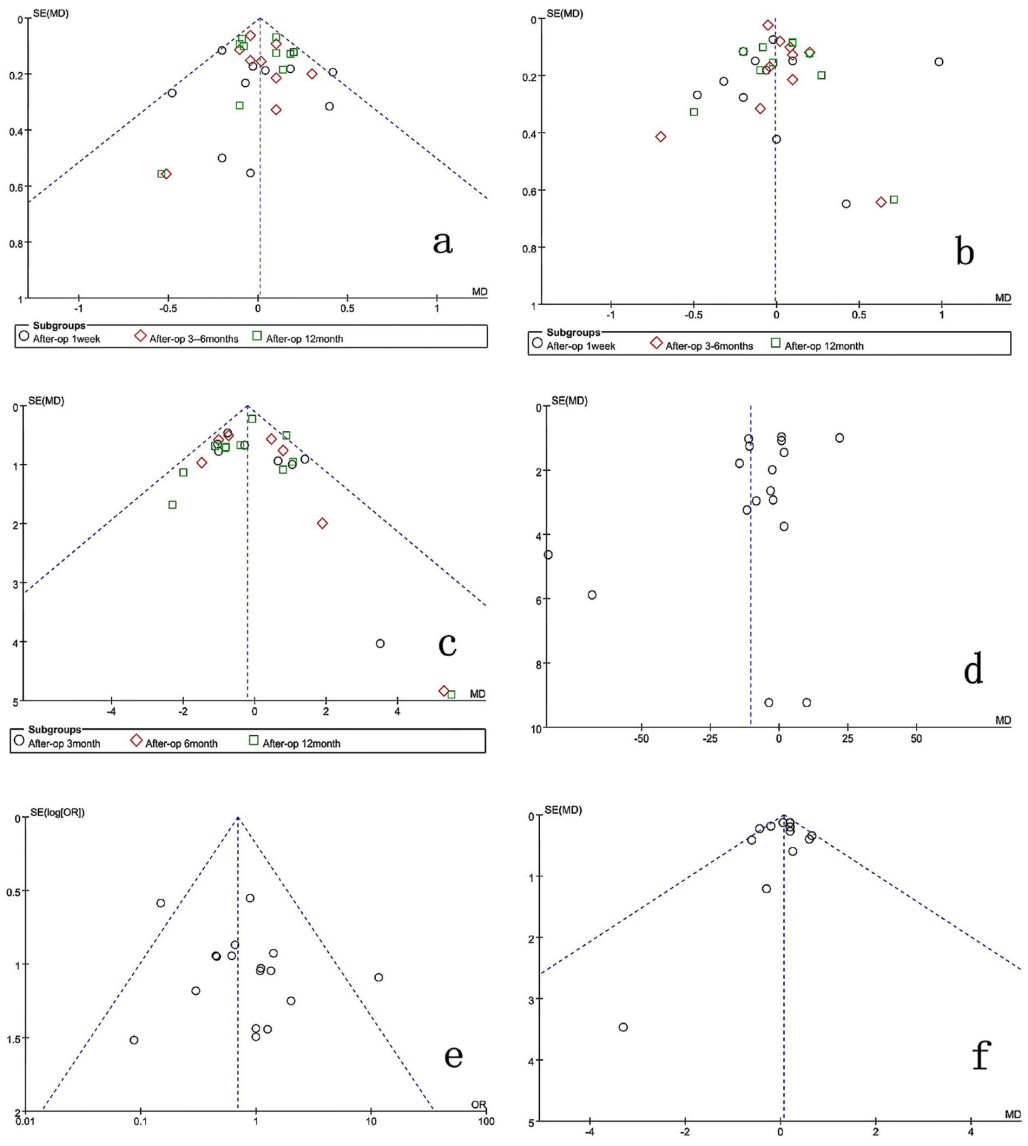
Funnel plot for publication bias of the studies included in the research. **(a)** Back-VAS; **(b)** leg-VAS; **(c)** ODI; **(d)** operation time; **(e)** complications; **(f)** hospitalization time.

Funnel plots were created with RevMan 5.3 software to investigate possible publication biases for primary outcomes. Asymmetric patterns observed in funnel plots for surgical duration, postoperative back pain VAS scores, lower limb pain VAS scores, and ODI scores suggest potential publication bias. Conversely, symmetric patterns emerged in funnel plots for postoperative complications and length of hospitalization, implying a lower probability of publication bias (Figure 12). Sensitivity analysis was conducted to verify the stability of results by sequentially excluding studies contributing markedly to heterogeneity (operation duration, VAS scores, intraoperative blood loss, dural expansion, facet joint resection angle). The minimal change in overall heterogeneity indicated stable and reliable findings. Variability among surgeons’ experience and patients’ individual pain tolerance levels could explain the observed heterogeneity.

## 4 Discussion

LSS is a common degenerative condition among middle-aged and elderly populations. Its incidence continues to rise with an increasing proportion of older individuals globally. The primary pathology involves degenerative changes and hyperplasia in structures around the spinal canal, particularly hypertrophy and ossification of facet joints and thickening of the ligamentum flavum (27, 28). Although conservative treatment may alleviate symptoms, surgical intervention is necessary in severe or refractory cases to enlarge the spinal canal and relieve nerve compression (29). Traditional open surgery achieves adequate decompression; however, it raises widespread concerns due to significant disruption to spinal anatomy, persistent postoperative pain, and muscle atrophy (30).

Currently, UE and UBE represent the most advanced minimally invasive spine surgery techniques [Notably, the Unilateral Endoscopy (UE) technique employs a single integrated metal cannula combining both the endoscopic lens and working channel to simultaneously visualize and manipulate the surgical site. In contrast, the Unilateral Biportal Endoscopy (UBE) technique utilizes two distinct ipsilateral portals - a dedicated viewing portal and a separate working portal - enabling completely independent observation and instrument manipulation during the procedure]. These approaches use smaller surgical incisions, lowering infection risks and minimizing muscle and ligament damage. Consequently, spinal structural integrity is better preserved, patients mobilize earlier, and recovery times shorten compared to traditional open surgery (31–33). However, previous studies reported that the limited working channel in UE restricts surgeons’ range of motion and instrumentation, increasing risks of inadequate decompression and dural injury, especially in severely degenerated spinal segments (34, 35). Due to its inherent dual-port design, the UBE technique effectively resolves visibility and mobility constraints encountered during surgery. Additionally, the independent and flexible working channels enable the use of open surgical instruments, reducing complications such as inadequate decompression and dural tears. With ongoing development and application, the indications for UBE will likely expand further.

This study demonstrated that the UBE approach was associated with significantly shorter operation times compared to UE. However, due to considerable heterogeneity, subgroup analysis was conducted according to study design. Prospective studies showed no significant difference between the two approaches, while retrospective studies indicated significantly shorter operation times in the UBE group. Thus, the apparent superiority of UBE in overall analysis appears primarily driven by retrospective studies exhibiting high heterogeneity. Prospective studies, considered more reliable, showed comparable operation times between the two techniques. The substantial heterogeneity suggests potential variations in patient selection, surgical technique, and outcome measurement among studies, as well as inherent biases in retrospective studies, such as selective reporting of favorable outcomes. Therefore, more prospective RCTs are required to confirm these findings.

Our team has conducted multiple clinical comparisons of these techniques. Based on our experience, proficient surgeons typically require shorter operative time for UBE procedures. Possible reasons include: (1) Independent working channels: UBE provides separate portals for instrumentation and visualization, increasing surgical flexibility; (2) Expanded instrumentation options: The technique accommodates various decompression tools, enhancing efficiency in removing hypertrophic bone and ligamentum flavum; (3) Technical limitations of UE: The single-channel cannula approach inherently restricts surgical field visualization and instrument maneuverability, prolonging operative time.

This study indicated favorable decompression outcomes from both surgical methods. Seven to eleven studies reported complete data on back VAS, leg VAS, and ODI scores. All scores improved significantly at final follow-up compared to preoperative values. The meta-analysis revealed no significant differences between the two groups regarding back VAS, leg VAS, and ODI scores at various follow-up intervals. These findings highlight the efficacy of both surgical approaches in alleviating preoperative pain, minimizing soft-tissue damage, preserving spinal muscles, promoting early patient mobilization, reducing complications associated with prolonged bed rest, and accelerating overall recovery.

In this meta-analysis, three studies reported intraoperative estimated blood loss, and eleven studies reported hospitalization duration. Results showed no significant differences between the two groups, consistent with findings from another meta-analysis (36). Both UBE and UE techniques utilize continuous saline irrigation to maintain clear visibility, raising the possibility of unrecognized intraoperative and postoperative hidden blood loss, which could bias outcomes. Moreover, few studies incorporated postoperative drain output into blood-loss calculations. Future studies should accurately quantify both visible and hidden blood loss, facilitating more precise assessments. Additionally, the seventeen included studies reported several surgical complications, including dural tears, cerebrospinal fluid leaks, epidural hematomas, and insufficient decompression. However, no significant differences in complication rates emerged between the two groups. Thus, UBE and UE techniques can both be considered relatively safe surgical options.

Among the 17 included studies, 9 reported dural sac areas before and after surgery. Both UBE and UE techniques significantly expanded dural area compared to preoperative measurements. However, only 7 studies provided explicit data on the magnitude of dural expansion (13, 16, 17, 19, 21, 22, 25). Meta-analysis demonstrated a significantly greater postoperative dural expansion in the UBE group. Additionally, four studies (11, 12, 18, 23) reported facet joint resection angles during surgery. Meta-analysis revealed no significant difference between the two surgical groups, further supporting that UBE achieves effective decompression without excessive resection of facet joints or adjacent vertebral structures. The authors suggest several possible reasons for this observation based on the literature: (1) UBE's independent working channels provide greater operational flexibility than UE; (2) UBE utilizes a wider variety of surgical instruments, facilitating decompression in difficult-to-reach areas and simplifying the removal of hypertrophic or ossified joint and ligamentous structures. Additionally, in clinical practice, our team has observed that UBE surgery provides a broader decompression range compared to UE.

This meta-analysis concludes that both UBE and UE effectively treat LSS, with comparable expansion of the spinal canal postoperatively. Due to its single operative channel, UE results in less muscle and soft tissue trauma, making it clinically acceptable to patients. However, the unique dual-channel approach of UBE offers greater surgical flexibility. With similar joint surface decompression, UBE provides a significantly larger dural expansion area. Furthermore, Liu (37) evaluated learning curves for UBE and UE techniques, concluding that for novices, UBE demonstrated better improvements in pain scores and fewer complications compared to UE (*p* < 0.05). Additionally, the learning curve threshold for UBE was reached earlier. However, for experienced surgeons, no significant difference was found (*p* > 0.05). Thus, based on current evidence, clinicians should preferentially consider UBE for patients with bilateral stenosis, severe spinal canal stenosis, or significant facet joint hypertrophy necessitating extensive decompression. For surgeons new to endoscopic procedures, beginning with UBE may reduce complications. Experienced surgeons can choose the appropriate technique based on personal preference and proficiency.

## 5 Limitations

This study has several limitations: (1) The majority of included studies are retrospective, which may introduce selection bias, inconsistent outcome measures, and underreporting of complications. Although retrospective studies provide valuable preliminary evidence, their inherent limitations weaken the robustness of conclusions compared to prospective designs. (2) The included studies predominantly originated from Asia (particularly China and South Korea), introducing potential regional bias. This reflects early adoption and rapid development of endoscopic spinal surgery in these regions, possibly limiting the generalizability of results due to differences in surgical indications, patient demographics, or healthcare systems. (3) Publication bias: Funnel plots for operation time, back VAS scores, leg VAS scores, and ODI scores exhibited asymmetric distributions, suggesting potential publication bias. Conversely, plots for complications and hospital stay appeared symmetrical. Publication bias may arise from preferential reporting of positive results, especially concerning novel techniques, and language restrictions (English and Chinese). Future research should include publications in additional languages to minimize bias. (4) Small sample sizes in individual studies and short follow-up periods (averaging only 6–12 months) limit long-term efficacy comparisons. Thus, larger, multicenter studies with extended follow-ups are needed. (5) Variations in surgical techniques and decompression assessments among surgeons and differences in patient pain perception contribute to significant study heterogeneity, potentially influencing outcomes. (6) Different follow-up intervals across studies may affect overall results.

Collectively, these limitations indicate that although this analysis represents the most comprehensive synthesis of UBE-ULBD vs. UE-ULBD to date, the current evidence remains at Level III (primarily retrospective comparative studies). Future multicenter, large-scale, prospective clinical trials with extended follow-ups are necessary to further clarify advantages and disadvantages between these endoscopic techniques in treating LSS.

## 6 Conclusions

In summary, current evidence indicates no significant differences between UBE and UE approaches for LSS in terms of intraoperative blood loss, hospital stay duration, pain scores, and complication rates. However, UBE demonstrates clear advantages regarding operative time and postoperative dural expansion (decompression effectiveness). Conversely, UE, with a single incision, provides advantages related to minimally invasive characteristics and reduced trauma. Therefore, in the future, clinicians should consider various factors when choosing between the two surgical options and provide more multicenter data to further clarify the advantages and disadvantages of each procedure. This will help establish a reasonable and standardized surgical approach for such diseases, minimize patient suffering, and promote recovery.

## Data Availability

The original contributions presented in the study are included in the article/Supplementary Material, further inquiries can be directed to the corresponding author.
